# Case Report: Short rib-thoracic dysplasia type 3 presenting with predominant respiratory manifestations

**DOI:** 10.3389/fped.2026.1958469

**Published:** 2026-09-21

**Authors:** Pei Tao, Zhigang Wang, Yinghong Fan, Wanmin Xia

**Affiliations:** Department of Pediatrics, Chengdu Women and Children’s Central Hospital, School of Medicine, University of Electronic Science and Technology of China, Chengdu, Sichuan, China

**Keywords:** case report, *DYNC2H1*, respiratory manifestations, short rib-thoracic dysplasia type 3, skeletal ciliopathy

## Abstract

Short rib-thoracic dysplasia type 3 (SRTD3) is a rare skeletal ciliopathy caused by pathogenic variants in *DYNC2H1*, with a broad spectrum of clinical severity ranging from lethal neonatal disease to mild phenotypes presenting later in childhood. We report a 20-month-old girl who presented with recurrent infection-associated wheezing and a rhinovirus-associated acute lower respiratory tract infection complicated by hypoxemia and respiratory distress. Chest computed tomography revealed bilateral patchy ground-glass opacities with focal consolidation and a mildly narrowed thoracic cage with shortened ribs, while skeletal radiographs demonstrated subtle shortening of the long bones and mild acetabular dysplasia. Prenatal ultrasonography did not reveal characteristic fetal skeletal abnormalities, and no prenatal genetic testing was performed. Postnatally, whole-exome sequencing was performed because of developmental delay and recurrent respiratory infections, identifying compound heterozygous *DYNC2H1* variants comprising a paternally inherited synonymous splice-altering variant and a large exon 1–88 deletion identified by WES-based copy-number analysis and reported as maternally inherited. Together with the characteristic radiographic findings, these genetic findings supported the diagnosis of SRTD3. This case illustrates that respiratory manifestations may attract clinical attention before the skeletal phenotype is recognized in mild SRTD3, particularly when skeletal abnormalities are subtle. It adds to the emerging spectrum of mild postnatal *DYNC2H1*-related SRTD3 and highlights the importance of integrating clinical, radiologic, and genetic findings in children with recurrent respiratory symptoms and subtle skeletal abnormalities.

## Introduction

Short rib-thoracic dysplasia (SRTD) comprises a group of rare autosomal recessive skeletal ciliopathies characterized by a narrow thoracic cage, shortened ribs, limb shortening, and variable respiratory compromise resulting from thoracic insufficiency. These disorders are caused by defects in primary cilia, which are essential for skeletal morphogenesis through multiple developmental signaling pathways, including Hedgehog signaling (1–3). Among the currently recognized subtypes, SRTD type 3 (SRTD3) is caused by pathogenic variants in *DYNC2H1*, which encodes the heavy chain of cytoplasmic dynein-2 required for retrograde intraflagellar transport and normal ciliary function (4).

The clinical spectrum of SRTD3 is remarkably heterogeneous. Severe cases are usually detected during the prenatal or neonatal period because of marked thoracic narrowing, severe skeletal abnormalities, and respiratory failure, whereas milder phenotypes may present later in infancy or childhood with subtle skeletal abnormalities that are easily overlooked (5). In milder cases, respiratory manifestations may attract clinical attention before the skeletal phenotype is recognized, particularly when thoracic and appendicular abnormalities are subtle.

Here, we describe a child with clinically, radiographically, and molecularly supported SRTD3 who came to clinical attention because of recurrent infection-associated wheezing and a rhinovirus-associated acute lower respiratory tract infection. This case highlights the diagnostic challenges of mild SRTD3 and the importance of integrating clinical, radiologic, and genetic findings when skeletal abnormalities are subtle.

## Case presentation

A 20-month-old girl was admitted with a 20-day history of persistent cough and a 10-day history of progressively worsening wheezing following an upper respiratory tract infection. Despite treatment at a local hospital, her respiratory symptoms continued to worsen.

She had experienced approximately 10 episodes of wheezing since infancy, typically following respiratory tract infections. Mild episodes improved with nebulized salbutamol and budesonide, whereas more severe episodes required hospitalization and additional systemic corticosteroid therapy. She had a history of infantile eczema, but peripheral blood eosinophil counts and total serum IgE levels were within the normal ranges; specific IgE testing had not been performed. There was no family history of asthma or other atopic diseases and no history of tobacco smoke exposure, aspiration, gastroesophageal reflux, or feeding-associated respiratory symptoms. No respiratory resuscitation was required at birth, and objective pulmonary function testing had not been performed because of her young age.

Developmental concerns became apparent at approximately 12 months of age and mainly involved gross motor and expressive language development. She sat independently at slightly over 8 months, crawled at slightly over 11 months, and stood independently at 12 months, but was unable to walk independently at 20 months. Expressive language was limited to a few simple syllables, while fine motor skills and language comprehension were reported to be relatively preserved. No records of a formal standardized developmental assessment were available.

Prenatal ultrasonography had not revealed characteristic fetal skeletal abnormalities, and no prenatal genetic testing had been performed. A chest radiograph obtained at 12 months showed no obvious rib abnormalities, and echocardiography at that time showed a small atrial septal defect (1.9 mm). At 18 months of age, because of persistent recurrent respiratory infections and developmental delay, skeletal radiographs were obtained and showed thoracolumbar kyphosis, abnormal morphology of several thoracolumbar vertebral bodies, and abnormal iliac morphology, raising suspicion of an underlying skeletal dysplasia. Blood samples for whole-exome sequencing (WES) were subsequently collected at the same age, and the genetic report became available at 19 months, identifying two *DYNC2H1* variants. However, a clinical diagnosis of SRTD3 had not been established before the current hospitalization. There was no family history of skeletal dysplasia or other hereditary disorders.

On admission, her temperature was 36.5 °C, heart rate was 158 beats/min, respiratory rate was 50 breaths/min, and oxygen saturation was 90% on room air, with intercostal retractions. Her weight was 8.0 kg (<−3 SD), length was 78 cm (approximately −2 SD), and head circumference was 45 cm (between −2 and −1 SD) according to the Chinese growth standards for age and sex (6). Arterial blood gas analysis showed a pH of 7.386, PaO_2_ of 60 mmHg, and PaCO_2_ of 38.2 mmHg. Physical examination revealed a mildly narrowed thoracic cage and broad, slightly shortened fingers, without polydactyly or obvious limb deformities. Bilateral wheezing and coarse crackles were heard on lung auscultation, predominantly over the right lung.

Chest computed tomography showed bilateral patchy ground-glass opacities with focal areas of consolidation, predominantly involving the right lung. A mildly narrowed thoracic cage and shortened ribs were also noted (Figures 1A–D). In view of these thoracic findings and the previous skeletal abnormalities, further skeletal evaluation was performed. Radiographs demonstrated mild acetabular dysplasia with delayed ossification of the femoral heads, relatively shortened long bones, and mild metaphyseal widening (Figures 2A–C).

**Figure 1:**
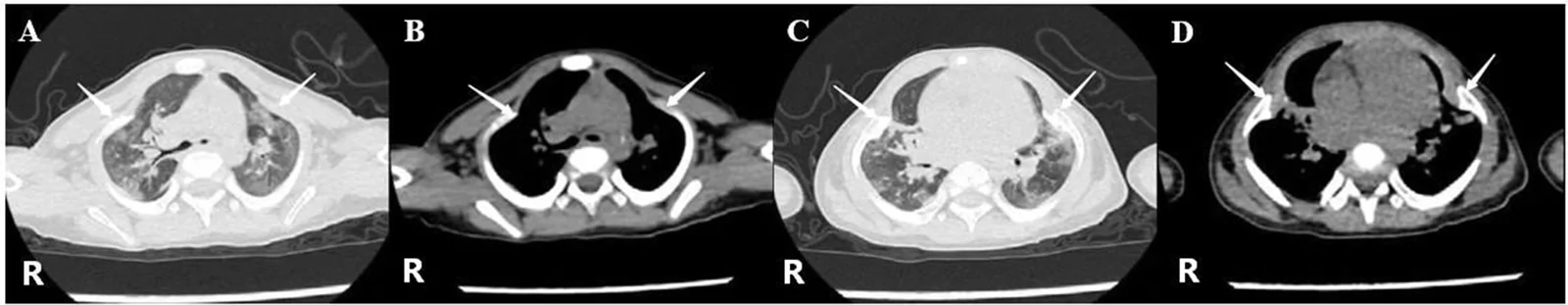
Chest computed tomography findings at presentation. **(A,C)** Lung-window images showing bilateral patchy ground-glass opacities with focal areas of consolidation, predominantly involving the right lung. **(B,D)** Mediastinal-window images showing a mildly narrowed thoracic cage with shortened ribs (arrows).

**Figure 2:**
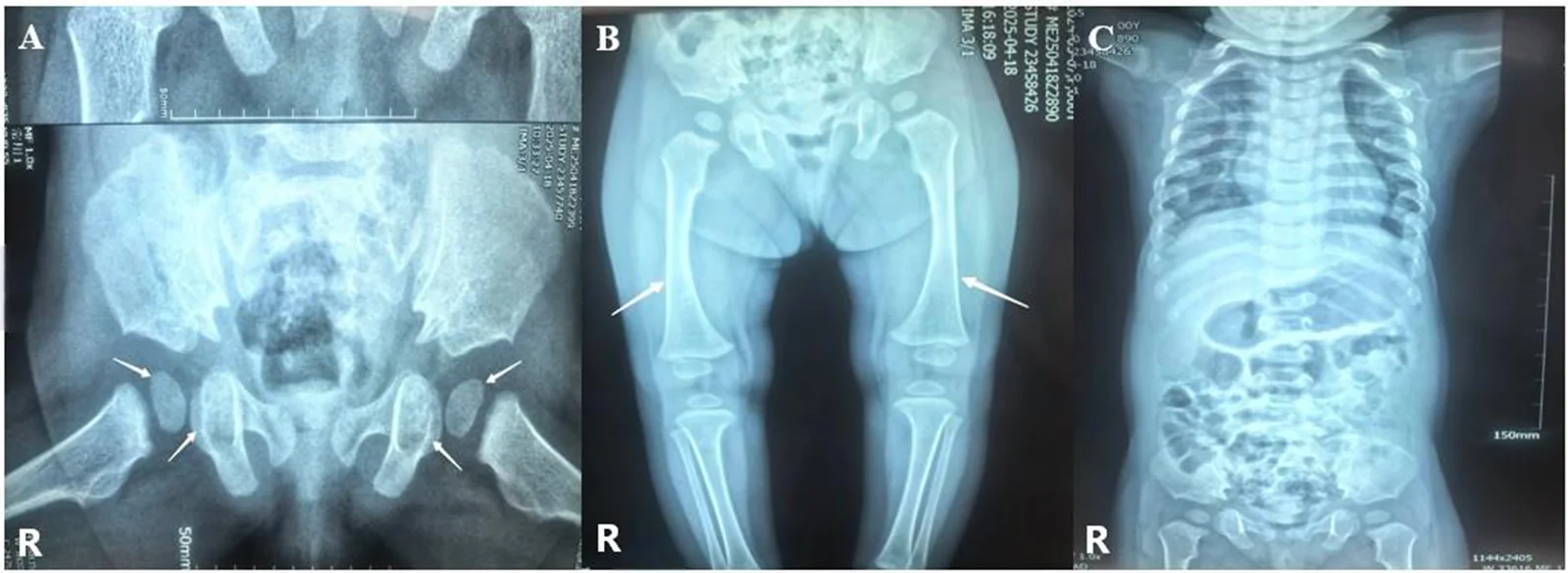
Skeletal radiographic findings in the patient with SRTD3. **(A)** Pelvic radiograph demonstrating mild acetabular dysplasia and delayed ossification of the femoral heads. **(B)** Radiograph of the lower extremities showing relatively shortened long bones. **(C)** Whole-body radiograph demonstrating a mildly narrowed thoracic cage and shortened ribs.

Laboratory investigations showed a white blood cell count of 14.43 × 10^9^/L, with 34.2% neutrophils and 52.1% lymphocytes, and a platelet count of 525 × 10^9^/L. High-sensitivity C-reactive protein was <0.50 mg/L, and procalcitonin was 0.18 ng/mL. Coagulation tests and renal biochemical parameters showed no clinically significant abnormalities. Blood culture was negative. A nasopharyngeal swab obtained at admission was positive for rhinovirus by polymerase chain reaction (PCR), with the result available on hospital day 2. Flexible bronchoscopy performed on hospital day 5 demonstrated diffuse tracheobronchial mucosal congestion with a small amount of airway secretions but no structural airway abnormalities. Rhinovirus was detected in bronchoalveolar lavage fluid, with a viral load of 4 × 10^5^ copies/mL and 32,538 sequence reads. No other clinically relevant pathogens were identified, and BAL culture was negative.

The WES performed before the current hospitalization identified compound heterozygous variants in *DYNC2H1* (NM_001080463.2). The c.11070G>A (p.P3690=) variant in exon 76 is a synonymous splice-altering variant previously shown to cause exon 76 skipping (7). It was rare in gnomAD (popmax allele frequency, 0.0000252678) and was classified as pathogenic according to ACMG/AMP criteria (PVS1, PM3_Strong, and PM2_Supporting), although ClinVar reported conflicting classifications of pathogenicity. Parental testing showed that the c.11070G>A variant was inherited from the father. The second variant was a heterozygous deletion involving exons 1–88 of *DYNC2H1*, detected by copy-number analysis of the clinical WES data. It was absent from gnomAD and classified as likely pathogenic (PVS1_Strong, PM2_Supporting, and PM3). The clinical report indicated that the deletion was inherited from the mother. The report stated that the detected variants underwent Sanger validation but did not specify how the exon 1–88 deletion or its maternal inheritance was confirmed. No breakpoint-junction assay or other orthogonal CNV confirmation was reported. The report did not provide exact genomic coordinates, breakpoints, estimated deletion size, CNV quality metrics, or information on neighboring-gene involvement, so the exact genomic extent of the deletion could not be determined. Together with the clinical and skeletal radiographic findings, these molecular findings supported the diagnosis of autosomal recessive short rib-thoracic dysplasia type 3.

The patient was initially supported with high-flow nasal cannula (HFNC) oxygen therapy at a flow rate of 8 L/min and an FiO_2_ of 0.50. She received nebulized terbutaline (2.5 mg twice daily) and budesonide (1 mg twice daily) for 10 days, intravenous methylprednisolone (2 mg/kg per dose every 12 h for 3 days, followed by 2 mg/kg once daily for 2 days), intravenous cefoperazone-sulbactam (50 mg/kg per dose every 8 h for 10 days), and nebulized recombinant human interferon-α1b (2 μg/kg per dose twice daily for 5 days). Her respiratory status gradually improved, and HFNC oxygen therapy was discontinued after 4 days. She was discharged in stable condition after a 10-day hospitalization. The clinical course from symptom onset to hospital discharge is summarized in Figure 3.

**Figure 3:**
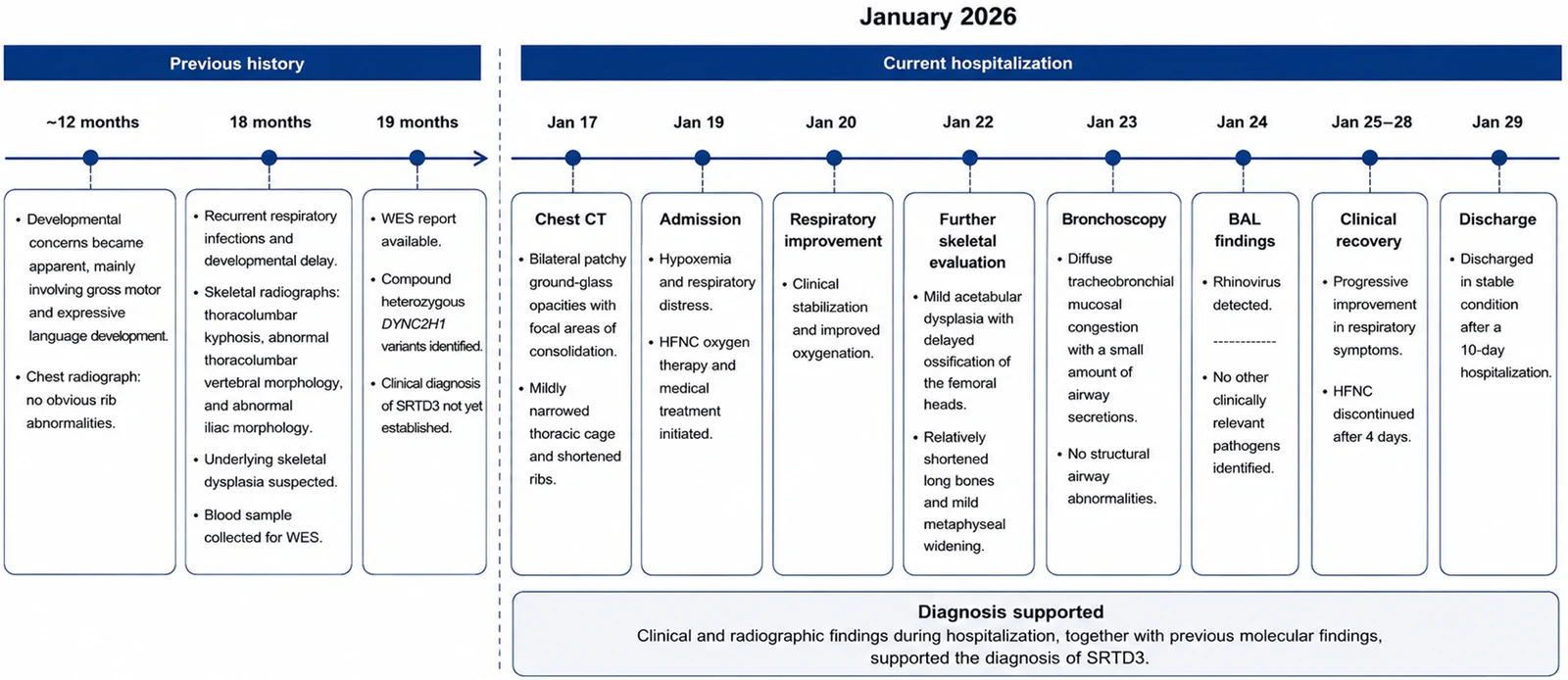
Clinical timeline of the patient. WES, whole-exome sequencing; *DYNC2H1*, dynein cytoplasmic 2 heavy chain 1; SRTD3, short rib-thoracic dysplasia type 3; CT, computed tomography; HFNC, high-flow nasal cannula; BAL, bronchoalveolar lavage.

At the 1-month follow-up visit, she remained asymptomatic without recurrent wheezing. Given the pulmonary parenchymal abnormalities observed during the acute illness, follow-up chest CT was performed to assess their resolution and reevaluate the thoracic abnormalities. Chest CT was selected rather than plain radiography because it allowed more detailed assessment of the previously observed ground-glass opacities, focal consolidation, and thoracic morphology. A pediatric low-dose CT protocol was used to minimize radiation exposure. Follow-up CT demonstrated near-complete resolution of the pulmonary parenchymal abnormalities, whereas the mild thoracic abnormality persisted (Figures 4A–H). The CT findings did not lead to any change in clinical management. During the subsequent 6-month follow-up period, she remained clinically stable without recurrent pneumonia, respiratory failure, or hospitalization. Given the relatively short follow-up period, no conclusion regarding long-term outcome can yet be made. Renal and hepatic ultrasonography and ophthalmological examination showed no abnormalities. Given her developmental delay and the potential multisystem involvement of SRTD3, multidisciplinary follow-up at 3-month intervals was recommended, including assessment of respiratory status, growth and development, renal and hepatic involvement, ophthalmological manifestations, and skeletal abnormalities. Follow-up with clinical genetics, orthopedics, and rehabilitation services was also recommended. Genetic counseling was provided regarding the autosomal recessive inheritance pattern, recurrence risk, and prenatal evaluation in future pregnancies.

**Figure 4:**
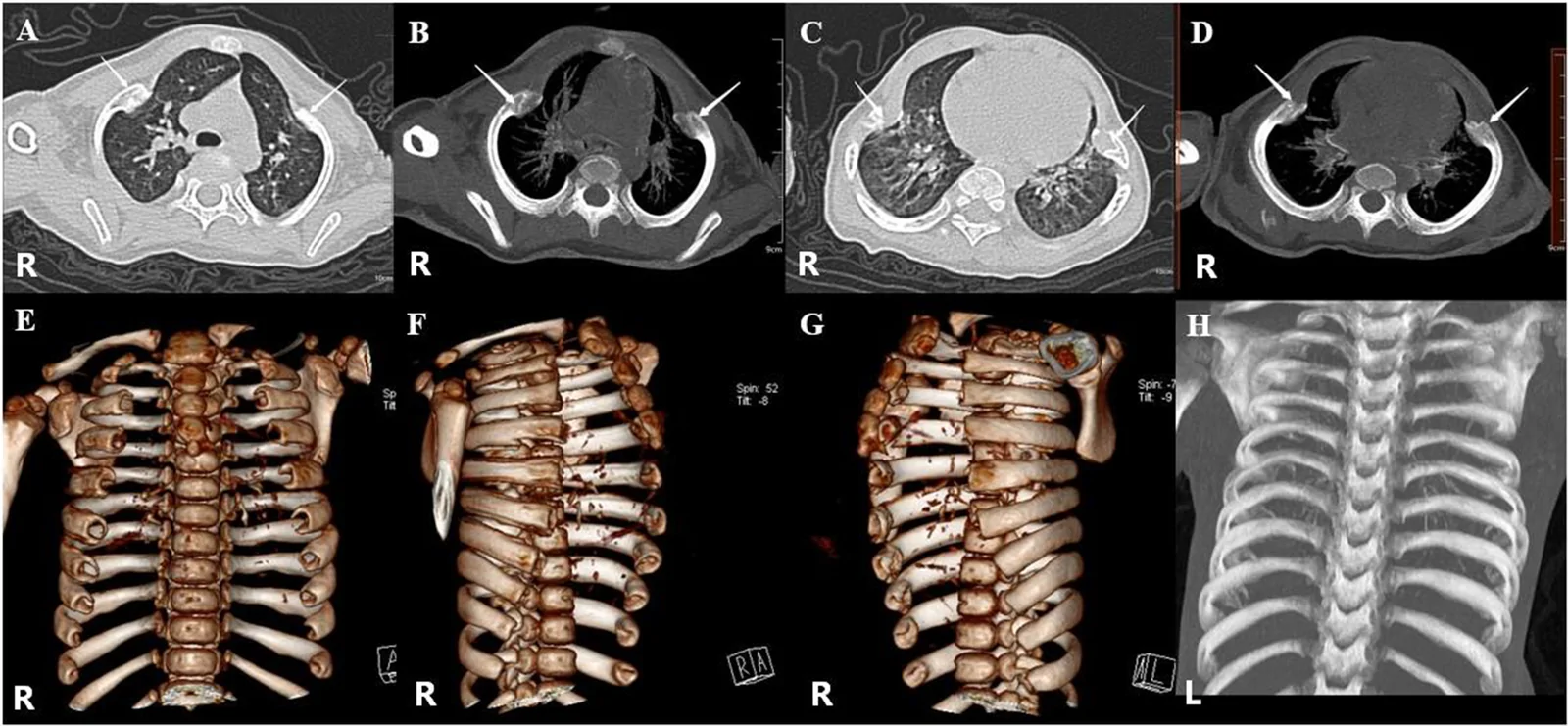
Follow-up chest computed tomography findings one month after discharge. **(A,C)** Lung-window images showing near-complete resolution of the previously observed ground-glass opacities and focal consolidation. **(B,D)** Mediastinal-window images, including a maximum intensity projection (MIP) image **(B)**, showing a persistently mildly narrowed thoracic cage with shortened ribs (arrows). **(E–G)** Three-dimensional reconstructions of the thoracic skeleton. **(H)** Coronal bone-window reconstructed CT image of the thoracic spine and rib cage.

## Discussion

Short rib-thoracic dysplasia type 3 (SRTD3) is a rare skeletal ciliopathy caused by pathogenic variants in *DYNC2H1*, which encodes the heavy chain of cytoplasmic dynein-2, a key component of the retrograde intraflagellar transport machinery (4). Disruption of dynein-2 function impairs ciliary transport and consequently affects skeletal development. Previous reports have shown that the clinical manifestations of SRTD3 range from severe prenatal skeletal dysplasia to relatively mild postnatal disease (3–5).

The marked phenotypic variability of SRTD3 poses a diagnostic challenge. Severe cases are usually recognized prenatally because of characteristic skeletal findings, including marked thoracic narrowing, short ribs, and limb abnormalities. In contrast, milder forms may lack obvious prenatal abnormalities and remain undiagnosed until infancy or childhood. In these patients, skeletal abnormalities may be subtle, while respiratory symptoms may become a more prominent clinical concern. Pattani et al. reported substantial phenotypic variability in nine patients with *DYNC2H1*-related SRTD, including four with mild disease and two requiring respiratory support (2). Their Case 5 carried the same c.11070G>A splice-altering variant identified in our patient and also had recurrent viral bronchiolitis, although these two cases alone do not establish a genotype–phenotype association. Emiralioglu et al. also described an infant with *DYNC2H1*-related Jeune syndrome who presented with recurrent pulmonary infections and respiratory distress (8). Selected published reports illustrating the clinical variability of *DYNC2H1*-related SRTD3 are summarized in Supplementary Table S1 (3–5, 8–15).

In the present case, prenatal ultrasonography did not reveal characteristic skeletal abnormalities. Postnatally, whole-exome sequencing was performed because of developmental delay and recurrent respiratory infections and identified compound heterozygous *DYNC2H1* variants. Subsequent radiographic evaluation revealed subtle skeletal abnormalities. Together, the genetic and radiographic findings supported the diagnosis of SRTD3. As no disease-specific treatment was available, clinical follow-up and supportive management were recommended. This case illustrates the diagnostic challenge of mild SRTD3 when prenatal skeletal abnormalities are not apparent and the postnatal skeletal phenotype remains subtle.

The acute respiratory deterioration and wheezing in this patient occurred in association with rhinovirus infection and improved with respiratory support and medical treatment. Follow-up imaging showed resolution of the acute pulmonary parenchymal abnormalities, whereas the thoracic abnormality persisted. Given the patient’s young age and the infection-associated pattern of wheezing, a direct causal relationship between recurrent wheezing and SRTD3 cannot be established. Although the underlying thoracic abnormality may have increased respiratory vulnerability during infection, the relative contributions of viral-triggered wheezing, airway dysfunction, and restrictive thoracic mechanics cannot be determined from this single case.

Compared with previously reported mild postnatal cases, our patient similarly presented with prominent respiratory manifestations and relatively subtle skeletal abnormalities. A distinctive molecular finding was the combination of the previously reported c.11070G>A splice-altering variant with a maternally inherited large deletion involving exons 1–88 of *DYNC2H1*. Our case further illustrates the clinical heterogeneity of mild *DYNC2H1*-related SRTD3 and the possibility that affected children may initially come to clinical attention because of respiratory symptoms. An underlying skeletal ciliopathy should therefore be considered in children with unexplained recurrent wheezing or respiratory infections when even mild thoracic or skeletal abnormalities are present. Integrating the clinical history with targeted radiologic and molecular genetic evaluation may help establish the diagnosis.

This case has several limitations. First, as a single-patient report, the findings cannot be generalized to the broader spectrum of *DYNC2H1*-related SRTD3. Second, the follow-up period was limited to 6 months, precluding conclusions regarding long-term respiratory, skeletal, and extraskeletal outcomes. Third, objective pulmonary function testing was not feasible because of the patient’s young age; therefore, the underlying respiratory physiology could not be characterized. Fourth, although recurrent wheezing was predominantly associated with respiratory tract infections, a causal relationship between SRTD3 and wheezing cannot be established from this case. Finally, the molecular findings have limitations. For the exon 1–88 deletion, the clinical genetic report did not provide exact genomic coordinates, breakpoints, estimated deletion size, CNV quality metrics, or information on neighboring-gene involvement, and no separate orthogonal confirmation was reported. Therefore, the precise extent of the deletion and any possible contribution of neighboring genes, including to the developmental phenotype, could not be determined.

In conclusion, respiratory symptoms may bring children with mild SRTD3 to clinical attention when the skeletal phenotype is not readily apparent. Recognition of subtle thoracic or skeletal abnormalities in this setting may prompt appropriate genetic evaluation and facilitate diagnosis.

## Data Availability

The original contributions presented in the study are included in the article/Supplementary Material, further inquiries can be directed to the corresponding authors.
