# Supplementary material for: Case Report: Short rib-thoracic dysplasia type 3 presenting with predominant respiratory manifestations
Source: Front Pediatr. 2026 Sep 21;14:1958469. doi: 10.3389/fped.2026.1958469 (PMC13635313; doi:10.3389/fped.2026.1958469)
Supplement: Supplementary file 1 [file Table1.docx]

Supplementary Table 1. Selected published reports of *DYNC2H1*-related short rib-thoracic dysplasia type 3 and comparison with the present case

| **Study** | **Age at**  **diagnosis** | ***DYNC2H1* variants** | **Skeletal phenotype** | **Respiratory manifestations** | **Outcome** |
| --- | --- | --- | --- | --- | --- |
| Mei et al. (2015) | Prenatal | Compound heterozygous | Short ribs, limb shortening | Severe thoracic insufficiency | Perinatal death |
| Okamoto et al. (2015) | Postnatal | Compound heterozygous | Severe skeletal dysplasia | Severe respiratory distress | Poor prognosis |
| Chen et al. (2018) | Prenatal | Compound heterozygous | Short ribs, narrow thorax, limb shortening | Severe thoracic insufficiency | Perinatal death |
| Deng et al. (2018) | Prenatal | Novel variants | Thoracic narrowing, skeletal dysplasia | Severe respiratory insufficiency | Poor prognosis |
| Emiralioglu et al. (2018) | Postnatal | *DYNC2H1* variants | Thoracic dystrophy | Recurrent pulmonary infections and respiratory distress | Not reported |
| Geng et al. (2020) | Prenatal | Compound heterozygous | Thoracic dysplasia, limb shortening | Severe respiratory insufficiency | Poor prognosis |
| Cheng et al. (2022) | Prenatal | Compound heterozygous | Short ribs, situs inversus | Severe respiratory insufficiency | Poor prognosis |
| Fang et al. (2023) | Prenatal/Postnatal | Compound heterozygous | Variable skeletal abnormalities | Moderate to severe respiratory involvement | Variable |
| Marshall et al. (2023) | Postnatal | Novel variants | Variable skeletal dysplasia | Variable respiratory manifestations | Variable |
| Asseri et al. (2024) | Postnatal | *DYNC2H1* variants | Mild thoracic dysplasia | Mild recurrent respiratory symptoms | Favorable |
| Pattani et al. (2025) | Prenatal/Postnatal | *DYNC2H1* variants | Narrow thorax with variable skeletal abnormalities | Variable respiratory manifestations | Variable |
| Present case | Postnatal | Compound heterozygous | Mild thoracic narrowing, subtle long-bone shortening, and mild acetabular dysplasia | Recurrent infection-associated wheezing and rhinovirus-associated lower respiratory tract infection complicated by hypoxemia and respiratory distress | Clinically stable during 6-month follow-up |
